# Investigation of the Relationship between Illogical Thoughts and Dependence on Others and Marriage Compatibility in the Iranian Veterans Exposed to Chemicals in Iran-Iraq War

**DOI:** 10.5539/gjhs.v6n5p38

**Published:** 2014-05-08

**Authors:** A. H. Afkar, M. Mahboubi, M. Neyakan Shahri, M. Mohamadi, F. Jalilian, F. Moradi

**Affiliations:** 1 Social Determinants of Health Research Center, Guilan University of Medical Sciences, Rasht, Iran; 2 Kermanshah University of Medical Sciences’, Kermanshah, Iran; 3 Substance Abuse Prevention Research Center, Kermanshah University of Medical Sciences, Kermanshah, Iran; 4 Emam Reza Hospital, Clinical Research Development, Kermanshah University of Medical Sciences, Iran

**Keywords:** marriage compatibility, illogical thoughts, dependence, chemical, veterans

## Abstract

**Background:**

Marital satisfaction is one of the main determinants of a family’s correct function. A large number of veterans have been reported to suffer from depression, anxiety, mood disorders, post-traumatic stress disorder, and physical disorders. The objective of this study is to examine association between Illogical thoughts and Dependence on Others and Marriage Compatibility in the Iranian Veterans Exposed to Chemicals in Iran-Iraq War.

**Methods:**

The present cross-sectional, analytical study was conducted on 200 veterans exposed to chemicals who were covered by the Foundation of Martyrs and Veterans Affairs, Gilangharb, Kermanshah, Iran. The study sample size was determined according to Krejcie and Morgan formula and the subjects were selected through random sampling. The study data were collected using marriage compatibility questionnaire, illogical thoughts questionnaire, and dependence on others questionnaire. The study data were analyzed using the SPSS statistical software (version18). Pearson correlation coefficient, multiple regression, and t-test were used in order to determine the relationships among the variables and compare the means.

**Results:**

The findings of the current study revealed no significant relationship between dependence on others, anxious attention, helplessness, avoiding problems, perfectionism, and autonomy and marriage compatibility. However, a significant relationship was found between failure and marriage compatibility.

**Discussion:**

Overall, the findings of the present study showed that the veterans of Gilangharb did not have disorders, but depended on others, particularly their spouses, due to their abnormal physical status. Sometimes, they cannot even do their personal tasks which results in their dependence on others eventually putting the veterans under pressure and stress.

## 1. Introduction

Family is the core of each society and the center for maintaining physical and mental health. It also plays a major role in raising the children or future mothers and fathers (Farjad, 1993). A healthy family is the one which efficiently adapts itself to cultural, environmental, psychological, social, and economic pressures at various stages of life (Navabinejad, 2004). Moreover, marital satisfaction is one of the main determinants of a family’s correct function. Marital satisfaction is in fact necessary for compatibility in marriage and continuation of effective relationships. Compatible couples are those with a high level of agreement who are satisfied with their relationships (Bernstein P. H. & Bernstein M. T., 1989). In order to have relationships with each other, the couples need to share their thoughts and emotions and show their appreciation (Soleiman, 2000).

Nonetheless, marriage among the healthy individuals is different from the veterans. Veterans have a large number of problems and, consequently, marriage is not quite easy for a veteran. In fact, most of the veterans are opposed to marriage due to their lack of financial and spiritual facilities (Esmaeilpour, 1991). Veteran’s Marriage problems are girl’s parents against, girl’s vague images of the life with veteran and Veterans’ mental state and physical. Also generally Veterans wives’ educations are higher than theirs (Shayan, 2007).

In psychiatry, thought disorder (TD) or formal thought disorder (FTD) refers to disorganized thinking as evidenced by disorganized speech. Specific thought disorders include poverty of speech, tangentiality, illogicality, perseveration, neologism, and thought blocking (Solomon, 2008).

Veterans exposed to chemicals are the victims of wars in which chemical warfare was used to fight against the enemies (Yekta, 2003). After the First World War, the widest chemical attacks occurred during Iran-Iraq war. United Nations reports have confirmed the usage of two important types of chemical agents; i.e., mustard gas and nerve agent, during this war (Veterans’ medical sciences and engineering research center, 2003). Other chemical agents, such as potassium cyanide and Feshren, were utilized, as well.

Unfortunately, a large number of veterans have been reported to suffer from depression, anxiety, mood disorders, post-traumatic stress disorder, and physical disorders (Khaghanizadeh & Sirati, 2004). Consequently, veterans experience a lot of problems in their marital life and cannot act as a normal individual in their relationships with their wives. Due to getting hurt during the chemical bombardment, such individuals experience problems in compatibility with their environment and this incompatibility is mostly manifested in their relationships with their family members, particularly their wives (Hemati, 2000). They have stated the most common problems to be sleeping disorders, headache, irritability, dizziness, amnesia, anxiety, and nightmare.

The studies conducted on the handicapped individuals and veterans and their social relationships showed that losing occupation highly affected their mental health and marital relationships. In addition, their children experienced behavioral changes mostly depression and educational failure (Yaghoubi Rad, 2003; Dejkam & Aminalroaya, 2003). In the same line, one study was performed on the wives and children of U.S. soldiers and the results showed that being jobless and staying at home disturbed the family plans and increased the tension among the family members (AVA report, U.S, 1980).

Furthermore, Bernstein and Goldenberg investigated the behavior of the wives of handicapped individuals and veterans regarding illogical beliefs, cognitive error, and unrealistic beliefs. They mentioned that the couples who continued their life with illogical thoughts and unresolved conflicts experienced considerable critical conflicts. These results can also be generalized to the wives of the veterans exposed to chemicals (Bernstein, 1989; Goldenberg, 2000).

Considering the role of the family, Thompson believes that a veteran’srelationship with his family and friends is an inseparable part of his life. Disability mostly affects the whole family, can have negative effects on the family’s integrity, and leads to ambiguities and conflicts in roles because the husband stays at home and the wife has to earn their living (Thompson, 1975).

After Iran-Iraq war, a large number of individuals in various parts of Iran underwent a lot of damages due to chemical bombardments. Gilangharb was one of the cities exposed to chemical bombardments during this war. Thus, physical as well as mental injuries were vastly detected in this area.

In spite of these individuals’ large number of problems, a limited number of studies have been conducted in this regard. Since most of the war veterans were young and had to live a long life with their new condition, understanding their mental, motivational, psychological, marriage, and familial problems are of utmost importance for enhancing their compatibility. However, due to the small number of researches conducted in this regard in Iran, therapists perform trial and error when providing services for these individuals. Iran - Iraq War occurred between 1980 to1988. Gilangharb is a border town that was vulnerable to enemy attack. Thus, the present study aims to investigate the veterans’ compatibility with their wives and dependence on others and some factors such as anxious attention, helplessness, avoiding problems, perfectionism, failure and autonomy affecting marriage compatibility.

## 2. Methods

The present cross-sectional, analytical study was conducted on 200 veterans exposed to chemicals who were covered by the Foundation of Martyrs and Veterans Affairs, Gilangharb, Kermanshah, Iran. The study sample size was determined according to Krejcie and Morgan formula and the subjects were selected through random sampling (Krejcie & Morgan, 1970). The veterans’ records included their problems regarding physical therapies, social works, and psychotherapy. Therefore, their problems regarding raising the children, marital satisfaction, spiritual and psychological affaires, educational affaires, etc. could be investigated.

The study data were collected using marriage compatibility questionnaire, illogical thoughts questionnaire, and dependence on others questionnaire.

Singh’s marriage compatibility questionnaire (1958) consists of two forms: for husbands and for wives. This questionnaire includes 10 yes/no questions. In addition, the scores range from 0 to 10. Scores 1 and 0 are assigned to positive and negative responses, respectively. The internal consistency of this questionnaire in a 75-subject sample was shown as 0.80-0.90. Besides, after interviewing 400 couples during more than 1 year, the questionnaire was shown to have desirable face and content validity (Ganji, 2005).

Jones’ illogical thoughts questionnaire included 100 closed-ended questions in 10 sections which were scored based on a 5-point Likert scale ranging from completely agree to completely disagree. Various studies have shown the questionnaire as a reliable one with Cranach’s alpha>0.7(Ebadi & Motamedi, 2005; Shirzadi, 2005).

The last questionnaire used in the study aimed to determine the dependence on others and reflected the respondents’ capabilities, emotions, and behaviors. This questionnaire was designed by Barret, Hirschfield, Goughand Klerman (Hirschfield et al., 1977). The dependence on others questionnaire included 48 questions answered through a 4-point Likert scale ranging from 1 (completely agree) to 4 (completely disagree) and higher scores showed higher dependence on others. The reliability of this questionnaire was reported as 0.7 through the test-retest method and 0.8 by correlating with a similar test. Its face and content validity was confirmed, as well. This study’s protocol was reviewed and confirmed by an external ethics board.

The study data were analyzed using the SPSS statistical software (v. 18). The relationship between disability percentage, dependence on others, anxious attention, failure, helplessness, avoiding problems, perfectionism, and autonomy and marriage compatibility was investigated. In addition, Pearson correlation coefficient, multiple regression, and t-test were used in order to determine the relationships among the variables and compare the means.

## 3. Results

The present study was conducted on 200 veterans exposed to chemicals including 102 males (51%) and 98 females (49%). The subjects’ age ranged from 25 to over 50 years and their mean age was 42 years.

By t-test determined that the mean difference between the male and female in terms of Dependence on others, Helplessness, Avoiding problems, Perfectionism and failure statistically were significant (Table 1). Due to the high sample size P values were significant and SD value was low.

**Table 1:** Mean and SD of the study variables based on sex

Variables	Group	Mean	SD	P-value
Dependence on others	Male	17.66	2.80	0.03
	Female	17.58	3.14	
Anxious attention	Male	18.42	2.90	0.12
	Female	18.54	3.05	
Helplessness	Male	19.08	3.11	0.019
	Female	19.19	3.51	
Avoiding problems	Male	17.37	2.87	0.024
	Female	17.76	2.99	
Perfectionism	Male	19.31	3.62	0.001
	Female	19.13	3.65	
Failure	Male	18.01	3.41	0.018
	Female	18.41	3.46	
Autonomy	Male	33.51	4.07	0.6
	Female	32.57	4.28	

As the table depicts, the highest means in both males and females were related to autonomy (33.51 and 32.57, respectively). On the other hand, the lowest mean was related to avoiding problems in males (17.37) and dependence on others in females (17.58). By t-test determined that the mean difference between the high age and low age in terms of Dependence on others, anxious attention, helplessness, Avoiding problems, Perfectionism and Autonomy statistically were significant. In this study, ages above 40 were considered as high ages and the rest were considered as low ages. The highest means in both high and low age groups were related to autonomy (32.87 and 33.14, respectively). On the other hand, the lowest mean was related to avoiding problems in high age groups (17.47) and dependence on others in low age groups (17.58) (Table 2).

**Table 2:** Mean and SD of the study variables based on age

Variables	Group	Mean	SD	P-value
Dependence on others	High age	17.71	3.12	0.01
	Low age	17.58	2.89	
Anxious attention	High age	18.71	2.97	0.035
	Low age	18.37	2.97	
Helplessness	High age	18.73	3.43	0.003
	Low age	19.33	3.24	
Avoiding problems	High age	17.47	3.19	0.002
	Low age	17.60	2.81	
Perfectionism	High age	19.95	3.70	0.016
	Low age	19.35	3.60	
Failure	High age	18.07	3.55	0.1
	Low age	18.27	3.39	
Autonomy	High age	32.87	4.67	0.015
	Low age	33.14	3.97	

In this study, more than 40% disability was considered as high disability percentage and the rest were considered as low disability percentage. The highest means were 34.23 and 32.77 in high and low disability percentage groups, respectively. On the other hand, the lowest means were 16.41 and 17.53 in high and low disability percentage groups, respectively (Table 3). Given that the high sample size SD value was low.

**Table 3:** Mean and SD of the study variables based on the disability percentage

Variables	Group	Mean	SD	P-value
Dependence on others	High disability percentage	18.02	3.37	0.001
	Low disability percentage	17.53	2.85	
Anxious attention	High disability percentage	18.56	2.37	0.18
	Low disability percentage	18.46	3.10	
Helplessness	High disability percentage	19.33	3.77	0.12
	Low disability percentage	19.09	3.19	
Avoiding problems	High disability percentage	16.41	2.20	0.032
	Low disability percentage	17.84	3.02	
Perfectionism	High disability percentage	18.61	3.74	0.025
	Low disability percentage	19.37	3.60	
Failure	High disability percentage	18.23	3.15	0.3
	Low disability percentage	18.20	3.50	
Autonomy	High disability percentage	34.23	3.70	0.002
	Low disability percentage	32.77	4.26	

The findings of the current study revealed no significant relationship between dependence on others, anxious attention, helplessness, avoiding problems, perfectionism, and autonomy and marriage compatibility. However, a significant relationship was found between failure and marriage compatibility by Pearson correlation coefficient (P=0.03) (Table 4).

**Table 4:** Correlation between marriage compatibility and other variables

Variables	Correlation coefficient	P-value
Dependence on others	0.097	0.17
Anxious attention	0.044	0.53
Helplessness	0.076	0.28
Avoiding problems	0.105	0.14
Perfectionism	0.127	0.07
Failure	0.153	0.03
Autonomy	-0.0127	0.07

Table 5 presents the summary of stepwise regression model. In this model, age, sex, disability percentage, illogical changes, and dependence on others were considered as predictor variables, while marriage compatibility was considered as the criterion variable. (Table5).

**Table 5:** Summary of stepwise regression model for predicting marriage compatibility based on age, sex, disability percentage, illogical changes, and dependence on others

Model	R	R^2^	Modified R^2^	∆R^2^	∆F	∆R
1	0.247	0.061	0.056	0.061	12.799	0.001
2	0.288	0.083	0.073	0.022	4.64	0.032

Model 1: predictor: sex- Model 2: predictor: dependence on others

Age, sex, disability percentage, illogical changes, and dependence on others are independent variables and marriage compatibility is dependent variable.

Sex explained 6.1% of marriage compatibility variance which was statistically significant (P=0.001). After entering the emotional dependence variable into the model, it explained 8.3% of marriage compatibility variance. (Table 6).

**Table 6:** Standard and non-standard coefficients in regression

Model	Non-standard coefficients (β)	Standard coefficients (B)	t	P-value
Constant value	14.02	-	24.74	0.001
Sex	0.555	0.26	3.78	0.001
Dependence on others	-0.026	-0.148	-2.15	0.0032

The most significant effect was related to sex (β=0.26) followed by dependence on others (β=-0.148). According to the results, female gender increased marriage compatibility. In addition, as the emotional dependence increased, marriage compatibility significantly decreased. The study findings revealed a significant correlation among the veterans’ and their wives’ anxiety, hostility, and phobic anxiety. However, no significant relationship was observed between age and disability percentage and marriage compatibility.

Considering the problems in various dimensions, 30%, 34%, and 36% of the veterans with above 70% disability were faced with the aforementioned problems at low, average, and high levels, respectively. Yet, after categorizing the problems, 26% of the veterans had little treatment problems, while 44% were faced with a lot of treatment problems. Besides, 46% of the veterans had serious social and cultural problems and 36.2% had a large number of economic problems.

The findings of the present study showed that the type of injury was effective in social problems; as the veterans suffered from more types of disabilities; they were faced with a larger number of social problems. Moreover, the number of years the veterans were covered by the Foundation of Martyrs and Veterans Affairs affected their personal and familial problems; the longer the veterans were supported by the organization, the less they were encountered with the above-mentioned problems. Residence status was also effective in the veterans’ problems. In case the veterans lived in their own houses rather than renting a house or living in their father’s house, they were faced with fewer economic problems.

## 4. Discussion

The present study aimed to investigate the relationship between marriage compatibility and illogical thoughts and dependence on others among the male and female veterans exposed to chemicals in Gilangharb, Iran. The study results revealed no significant relationship between marriage compatibility and dependence on others. This is in contrast with the study by Thompson showing the veterans’ relationship with their spouses and families as an inseparable part of their lives (Thompson, 1975). Due to the cultural similarities of the study society, familial relationships, and high interactions with others in this region, dependence on others is observed even in daily activities, which sometimes leads to reduction of marriage compatibility.

In this study, no significant relationship was observed between marriage compatibility and anxious attention, which is on the contrary to the results of the studies by Bernstein and Goldenberg (Bernstein, 1989; Goldenberg, 2006). They assessed the behavior of the veterans’ wives with respect to illogical beliefs and stated that the couples continuing their lives with illogical thoughts experienced conflicts. The difference between the present study and those performed by Bernstein and Goldenberg might be due to the cultural status of the societies under study (Bernstein, 1989; Goldenberg, 2006). In the society under the current investigation, due to adherence to traditions, the individuals are not worried about losing theirspouses’ attention and this increases their marriage compatibility.

In this study, no significant association was found between marriage compatibility and helplessness for change, which is in contrast with the results of the study by Ellis (Ellis, 1989). He believed that using the cognitive-emotional treatment method, the individuals tend to think and act logically and, consequently, their marriage compatibility increases. It should be mentioned that the society under the present study was a traditional one in which the general tendency toward religion is strongly emphasized. Since any change among the adherent individuals to tradition is very difficult, marriage compatibility is increased.

The findings of the present study showed no significant relationship between avoiding problems and marriage compatibility, which is not in agreement with any of the studies conducted on the issue. This reveals the sufficient satisfaction in the study population. They receive their income from the government and do not expect much. Therefore, they try to avoid problems and not to interfere in others’ affaires. Thus, it can be concluded that the couples solve their own problems which leads to increase in marriage compatibility.

In the current study, no significant relationship was observed between marriage compatibility and perfectionism. It should be noted that perfectionism in the society under investigation has its roots in religious beliefs and reveals adherence to traditions. Therefore, perfectionism strengthens marriage compatibility because marriage compatibility and respect to spouse are highly admired in religious beliefs.

The present study results revealed a significant relationship between marriage compatibility and failure. The veterans under the present study avoided problems and rejected any change which was against their tradition. Otherwise, they would experience failure which has negative impacts on the couples’ relationships.

In this study, no significant relationship was observed between marriage compatibility and autonomy. Similar results were observed in another study (Ebstein et al., 2005). In the present study society, culture is highly taken into account. Due to the cultural status of the society, autonomy and independence has grown among the individuals and they make their decisions autonomously. Thus, the extent to which the spouse is involved in decision making affects marriage compatibility.

In one study conducted on the relationship between illogical thinking and dependence on others and marriage compatibility among non-veteran couples in Mashhad, marriage compatibility was similar in the two sexes. However, the findings of the present study revealed a significant difference between the two sexes regarding marriage compatibility (Momenzadeh, Mazaheri, & Heidari, 2005).

The studies performed on the veterans’ marriage compatibility in Iran have shown losing occupation as one of the main factors affecting marriage compatibility as well as social and psychological problem (Momenzadeh, 2005; Sadeghi Fasaee, 2013; Dejkam, 2003).

Overall, the findings of the present study showed that the veterans of Gilangharb did not have disorders, but depended on others, particularly their spouses, due to their abnormal physical status. Sometimes, they cannot even do their personal tasks which results in their dependence on others eventually putting the veterans under pressure and stress.

### 4.1 Limitations of the Research

Some information could not be accessed because of being secret and most of the information was collected based on the memories of war. These are cross sectional data and the measures used were dated. Iran- Iraq war began about 34 years ago. Moreover, due to the small number of chemical wars compared to other types of wars, a limited number of studies have been conducted on this issue and we faced a great difficulty in reviewing the literature.

### 4.2 Suggestions

- Supporting the veterans both emotionally and psychologically
- Providing the veterans with occupations according to their disability percentage
- Supplying facilities including transfer
- Appreciating the veterans’ efforts and considering facilities and rewards for them
- Timely hospitalization of the veterans and providing them with medication
- Since the veterans have been forgotten in some dimensions of the society, it is necessary to provide a condition through advertisement and culturalization so that the veterans would not feel unsupported.

## References

[ref1] Bernstein P. H., Bernstein M. (1989). Recognition and treatment of marital conflict. (HR. Sohrabi, Trans).

[ref2] Dejkam M., Aminalroaya A. (2003). Comparison of Psychiatrics veterans’ wives mental health referred to Sadr hospital with wives of mental illness referred to Imam Hossein Hospital. The first Scientific Veterans and Families Conference.

[ref3] Ebadi Gh, Motamedin M. (2005). Factor structure of Irrational Jones test on Ahvaz. Journal of Psychology.

[ref4] Elis A. (1989). Rational- emotive couples’ therapy.

[ref5] Epstein N. B., Chen F., BeyderKamjou I. (2005). Relationship standard and marital satisfaction in Chinese and American couples. Journal of marital and family therapy.

[ref6] Esmaeilpour M. M. (1991). Second life.

[ref7] Farjad M. H. (1993). Social pathology of family conflict and divorce.

[ref8] Ganji H. (2005). Personality assessment.

[ref9] Goldenberg L., Goldenberg H. (2000). Psychology. 492 pages.

[ref10] Goldenberg L., Goldenberg H., Hoseinshahi H., Naghshbandi S., Arjmand A. (2006). Family therapy.

[ref11] Hemati M.A. (2000). Neuropsychiatric complications caused by the war.

[ref12] Hirschfield M. A., Klerman G. L., Gouch H. G., Barrett J., Korchin Sh J., Chodoff P. (1977). A measure of interpersonal Dependence. Journal of personality Assessment.

[ref13] Khaghanizade M., Sirati M. (1383). Effect of personal, family, economic and social agents to intensive of symptom in veterans. Military of medicine.

[ref14] Krejcie R. V., Morgan D. W. (1970). Determining sample size for research activities. Educational and Psychological Measurement.

[ref15] Momenzadeh F., Mazaheri M. A., Heidari M. (2005). Irrational thinking and attachment patterns associated with marital compatibility. Journal of Family Research.

[ref16] Navabinejad S. H. (2004). Marriage counseling and Family Therapy.

[ref17] SadeghiFasaee S., Erfanmanesh I. (2013). Sociological analysis of the impact of modernization on Iranian families and the need to drafting Islamic Iranian pattern. Journal of Women in Art.

[ref18] Shayan F. (2007). Studied the problems of spinal cord injured spouse. Resalat, (6280), 19.

[ref19] Shirzadi S. (2005). The role of self-concept emerged in the relationship between depression and irrational in a group of high school students. (Unpublished MSc Thesis). Faculty of Education and Psychology, ShahidBeheshti University, Tehran, Iran.

[ref20] Soleiman A. (2000). The effect of marital dissatisfaction and irrational thoughts. (Unpublished MScThesis). Teacher Training University of Tehran, Iran.

[ref21] Solomon M., Ozonoff S., Carter C., Caplan R. (2008). Formal thought disorder and the autism spectrum: relationship with symptoms, executive control, and anxiety. J. Autism. Dev. Disord.

[ref22] Thompson R. F. (1975). Introduction to Physiological Psychology. California.

[ref23] Veterans’medical sciences and engineering research center (2003). Health guide.

[ref24] Yaghoubi Rad S. (2003). Determine the type and extent of veterans injury problems (70% and above) in Kerman.

[ref25] Yekta H. (2003). Use of chemical weapons in the Iran-Iraq war. Journal of Negin Iran.

